# *Aedes fluviatilis* cell lines as new tools to study metabolic and immune interactions in mosquito-Wolbachia symbiosis

**DOI:** 10.1038/s41598-021-98738-7

**Published:** 2021-09-28

**Authors:** Christiano Calixto Conceição, Jhenifer Nascimento da Silva, Angélica Arcanjo, Cíntia Lopes Nogueira, Leonardo Araujo de Abreu, Pedro Lagerblad de Oliveira, Katia C. Gondim, Bruno Moraes, Stephanie Serafim de Carvalho, Renato Martins da Silva, Itabajara da Silva Vaz, Luciano Andrade Moreira, Carlos Logullo

**Affiliations:** 1 Laboratório de Bioquímica de Artrópodes Hematófagos, Laboratório de Bioquímica e Fisiologia de Insetos – IBqM and Laboratório Integrado de Bioquímica Hatisaburo Masuda – NUPEM, Universidade Federal Do Rio de Janeiro - UFRJ, Av. Carlos Chagas Filho, 373, bloco D, subsolo, sala 05. Prédio Do CCS. Ilha Do Fundão, Rio de Janeiro, RJ 21941-902 Brazil; 2 Centro de Biotecnologia and Faculdade de Veterinária, Universidade Federal Do Rio Grande Do Sul - UFRGS, Porto Alegre, RS Brazil; 3 Instituto Nacional de Ciência E Tecnologia Em Entomologia Molecular, Rio de Janeiro, RJ Brazil; 4 Grupo Mosquitos Vetores: Endossimbiontes e Interação Patógeno Vetor, Instituto René Rachou - Fiocruz, Belo Horizonte, Minas Gerais Brazil

**Keywords:** Carbohydrates, Glycobiology

## Abstract

In the present work, we established two novel embryonic cell lines from the mosquito *Aedes fluviatilis* containing or not the naturally occurring symbiont bacteria Wolbachia, which were called *w*Aflu1 and Aflu2, respectively. We also obtained wAflu1 without Wolbachia after tetracycline treatment, named wAflu1.tet. Morphofunctional characterization was performed to help elucidate the symbiont-host interaction in the context of energy metabolism regulation and molecular mechanisms of the immune responses involved. The presence of *Wolbachia pipientis* improves energy performance in *A. fluviatilis* cells; it affects the regulation of key energy sources such as lipids, proteins, and carbohydrates, making the distribution of actin more peripheral and with extensions that come into contact with neighboring cells. Additionally, innate immunity mechanisms were activated, showing that the *w*Aflu1 and wAflu1.tet cells are responsive after the stimulus using Gram negative bacteria. Therefore, this work confirms the natural, mutually co-regulating symbiotic relationship between *W. pipientis* and *A. fluviatilis*, modulating the host metabolism and immune pathway activation. The results presented here add important resources to the current knowledge of Wolbachia-arthropod interactions.

## Introduction

Bacteria of the genus Wolbachia are a powerful model for host-microbe interaction studies^1^. Genomic analysis suggests that Wolbachia lacks a series of essential enzymes, including part of the glycolytic pathway, which indicates that the bacterium relies on its host cell machinery for energy production and other metabolic functions^2–5^. The obligate symbiosis between insects and bacteria are among the most complex associations; host and symbiont are integrated into a tidy biological entity and are unable to survive otherwise^6,7^. Various endosymbiotic bacterial species are common in arthropods, complementing their often specialized nutrition, aiding compound detoxification, and maintaining immune homeostasis^8–12^.

*Wolbachia pipientis* is a maternally transmitted bacterium that colonizes 40% of arthropods and about 66% of insects^1,13^, as well as filarial nematodes and terrestrial crustaceans^1,14^. The transinfection of the bacterium Wolbachia into insect hosts has been recently performed in order to elucidate the mechanisms of host-parasite interaction and potentially identify control alternatives for insect-borne diseases^15^. Although it has not been possible to grow Wolbachia in a cell-free system for genetic manipulation, Wolbachia transinfections have been used as strategies in public health programs due to its ability to reduce the transmission of some disease-causing pathogens, especially those transmitted by mosquitoes such as Dengue and Zika fever^16–18^.

In contrast to *Aedes aegypti*, where Wolbachia has been artificially microinjected into embryos^19–21^, the mosquito *Aedes fluviatilis* is naturally infected by the *w*Flu strain, an ancient, natural endosymbiotic relationship where the bacteria don’t seem to interfere on the insect’s fitness^8,22,23^. It is well established that Wolbachia, as an obligate intracellular bacterium, can exhibit symbiont, parasitic, mutualistic, or commensal relationships with diverse insects, potentially affecting host fitness and/or causing reproductive aberrations to enhance their own transmission^1,24^. Despite possible effects on reproduction (e.g. cytoplasmic incompatibility, parthenogenesis, male-killing, feminization of genetic male)^25^, they don’t always account for Wolbachia wide distribution in nature, since in many cases this genus does not interfere with host reproduction^26–29^.

Research efforts have yielded important insights into the mechanisms by which Wolbachia alters host biology, but progress has been slow^18^. Also, the bacterial factors that interact with the host have remained largely elusive, and these factors can vary depending on whether the bacteria are acquired naturally or artificially. Therefore, new models to study the interaction between Wolbachia and its hosts can shed new light on the biological mechanisms of this complex relationship.

In the present work, two novel *Aedes fluviatilis* embryonic cell lines were established and characterized. The study outlines the contribution of *Wolbachia pipientis* to *Aedes fluviatilis* metabolism and immune response. This new cell system is also an important tool for researchers investigating the host-symbiont metabolic regulatory networks and molecular mechanisms involved in insect-Wolbachia interactions.

## Results

### Characterization of Aedes fluviatilis embryonic cell lines wAflu1 and Aflu2

**Figure 1:**
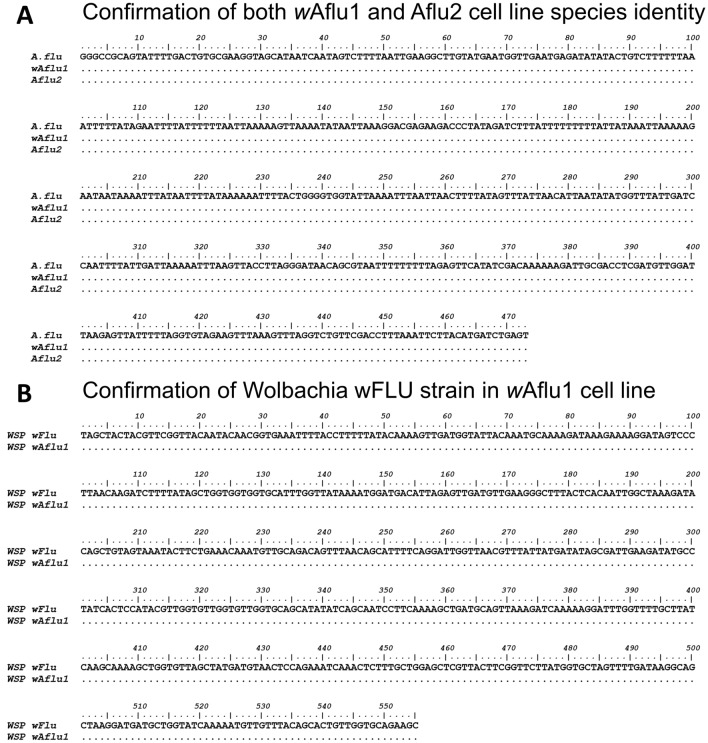
Sequence identification of mosquito cell lines and the Wolbachia strain. Sequence alignment of the 16S rRNA fragment obtained from the *Aedes fluviatilis* mosquito (MW574133) with 16S rRNA fragment obtained from the *w*Aflu1 and Aflu2 cell lines, showing identical sequences (**A**). Sequence alignment of WSP gene fragment obtained from Wolbachia strain *w*Flu (GQ917108) is identical to the sequence amplified from *w*Aflu1 cell line (**B**). Dots represent identical nucleotides.

**Figure 2:**
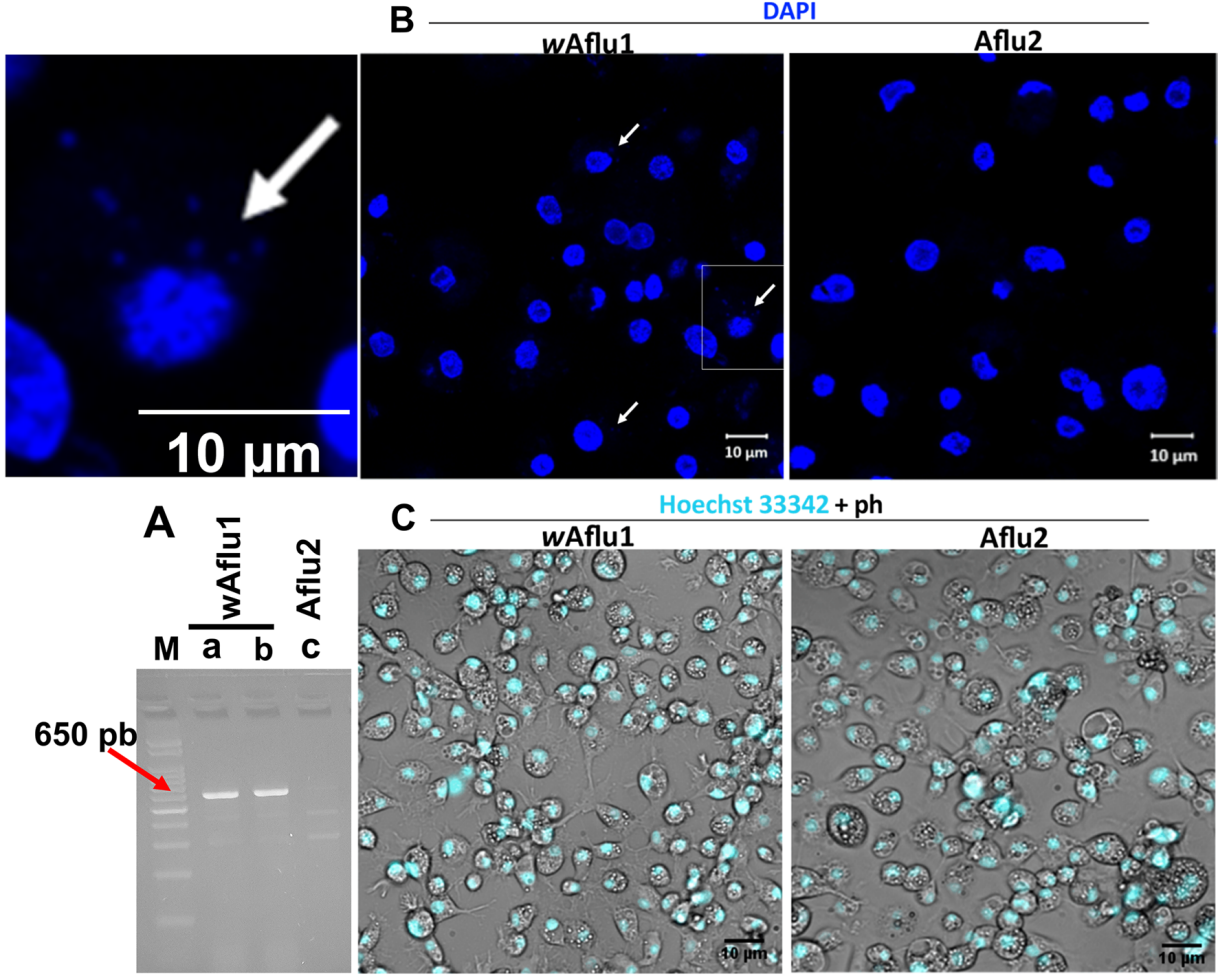
*Aedes fluviatilis* embryonic cell lines *w*Aflu1 and Aflu2. Embryonic cell line *w*Aflu1 was positive for the Wolbachia-specific gene, WSP, which was absent in Aflu2 cells (**A**, lane a and c, respectively). Amplification of WSP gene transcripts was still detected in *w*Aflu1 cells after cryopreservation (**A**, lane b). The WSP fragment amplified by RT–PCR has about 650 pb, as indicated by 100 pb marker (**A**, lane M). *w*Aflu1 and Aflu2 nuclei were visualized using DAPI staining and laser scanning confocal microscope Zeiss LSM 710, with the presence of Wolbachia being also observable in the cytosol of *w*Aflu1 cells, as indicated by the white arrow in the left panel enlarged from the highlighted quadrant (**B**). Additionally, nuclei of live cells were stained with Hoechst and visualized by fluorescence microscopy, here merged with phase contrast (ph) images to show overall cell morphology, with granular vesicles in the cytosol (**C**).

Partial sequencing of the mitochondrial 16S rRNA gene confirmed the cell lines belonged to *Aedes fluviatilis* species. *w*Aflu1 and Aflu2 both showed complete identity with deposited transcriptomic 16S rRNA sequence (MW574133) from *A. fluviatilis* (Fig. 1A). Also, the Wolbachia strain (*w*Flu) present in *w*Aflu1 cell line from naturally infected embryos had its identity confirmed by partial sequencing of WSP gene. Its showed complete identity with deposited (GQ917108) (Fig. 1B). The presence of Wolbachia in *w*Aflu1 cell line was confirmed by the amplification of a ~ 650-bp fragment by RT-PCR using WSP primers (Fig. 2A, lane a). Also, it was shown that Wolbachia remains viable after recovery of cryopreserved *w*Aflu1 cells (Fig. 2A, lane b). No PCR product was observed in the same analysis of Aflu2 cells, confirming the absence of Wolbachia in this cell line (Fig. 2A, lane c). Also, no PCR product was observed in the same analysis of wAflu1.tet, confirming the absence of Wolbachia by tetracycline treatment (Supplementary Figure 1).

**Figure 3:**
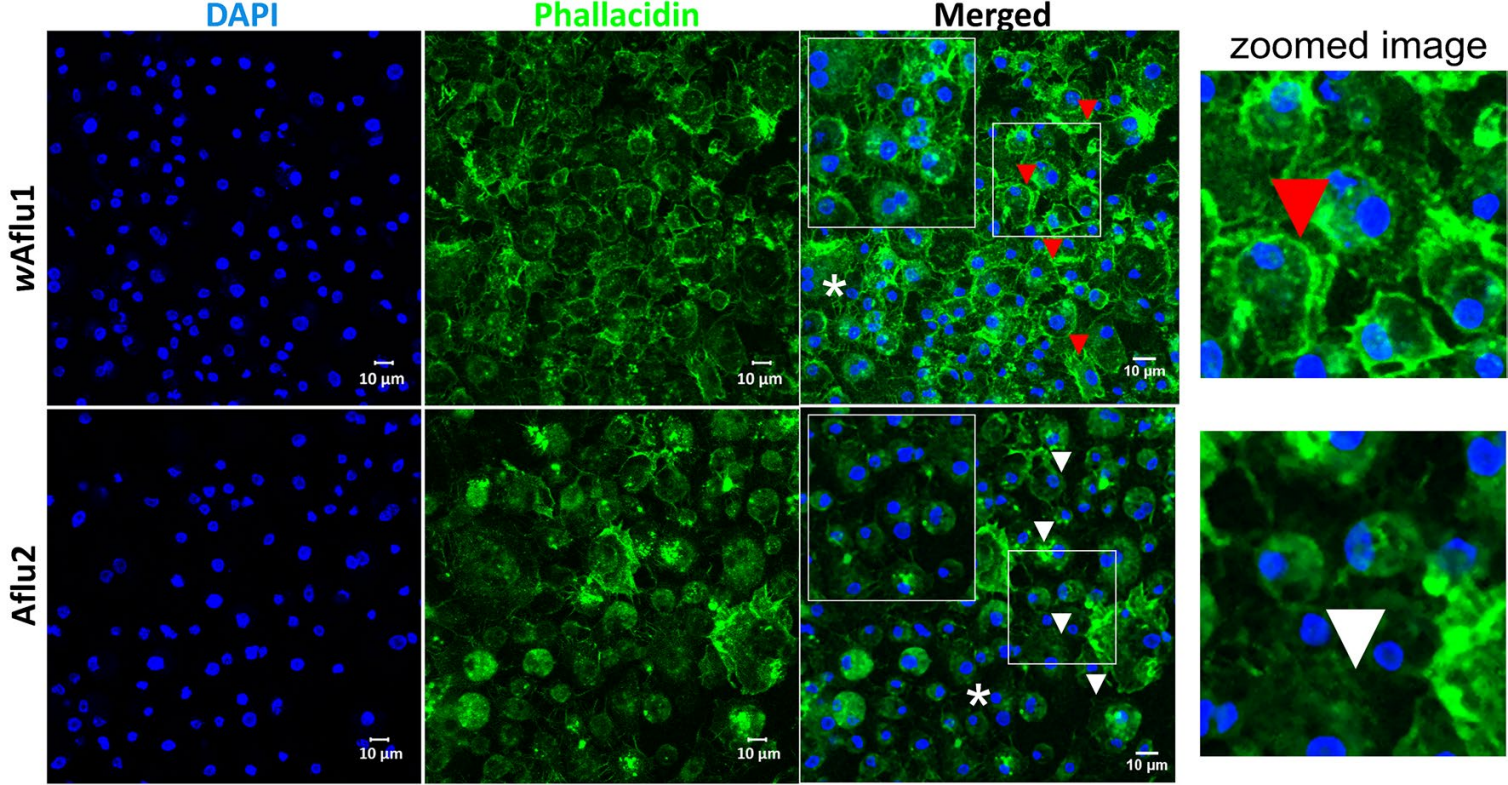
*w*Aflu1 has a more peripheral actin distribution than Aflu2. *w*Aflu1 and Aflu2 cell morphology was observed under a confocal laser scanning microscope, Zeiss LSM 710. Actin cytoskeletal morphology (green) and nuclei (blue) were observed three days after subculture using Phallacidin and DAPI staining, respectively. wAflu1 cell line has more peripheral actin distribution (red arrow head and detailed view in zoomed image) than Aflu2 (white arrow head). Scale Bar: 10 µm.

*w*Aflu1 and Aflu2 cells grow attached to the surface and they are morphologically similar, with a vast majority of round cells and some cells with fusiform-like appearance, as observed by phase contrast microscopy (Fig. 2C), with Aflu2 line presenting a generally higher proportion of round cells than *w*Aflu1. Both cell lines also showed well defined nuclei (observed with DAPI in Fig. 2B) of variable size relative to the cytoplasm (observed with Hoechst in Fig. 2C), and slightly larger cells with cytoplasmic vesicles (phase contrast images in Fig. 2C). In addition, *w*Aflu1 cells presented more organized actin filaments on the periphery (Fig. 3, red arrows, zoomed image and Supplementary Figure 2), forming extensions that contact neighboring cells (Fig. 3, magnified white asterisk region in top merged panel), while Aflu2 presented more diffuse actin (Fig. 3, white arrows) and fewer extensions (Fig. 3, magnified white asterisk region in lower merged panel).

### Cell line with or without Wolbachia have different metabolic features

**Figure 4:**
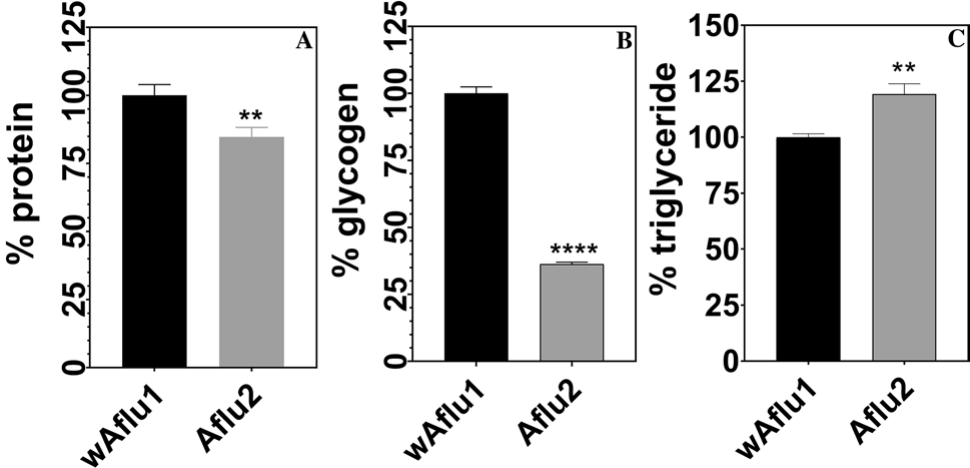
Major energy sources in *Aedes fluviatilis* cell lines. *w*Aflu1 cell line has higher protein and glycogen contents in comparison to Aflu2 cell line (**A**, **B**), while Aflu2 cells have more lipids (Triglycerides) than *w*Aflu1 (**C**). The experiments were performed with three independent biological samples in three experimental replicates each, **p < 0.01; ****p < 0.0001, using student’s t-test.

**Figure 5:**
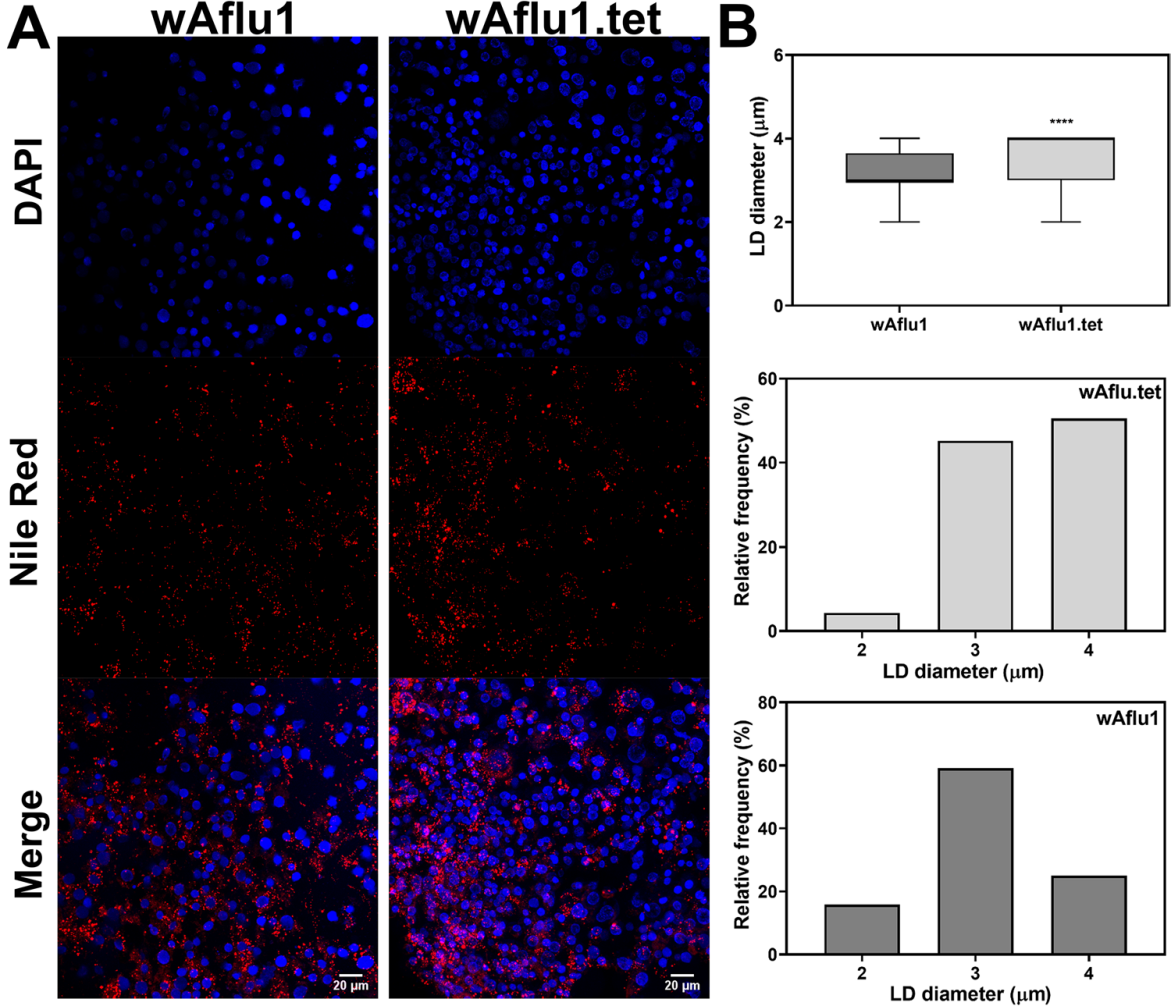
wAflu1 cells have smaller lipid droplets lipid droplets than *w*Aflu1.tet. Cells were stained with Nile Red and DAPI and observed under a laser scanning confocal microscope (**A**). wAflu1 cells show smaller lipid droplet (LD) average diameter and higher relative frequency of smaller LD, while wAflu1.tet has higher relative frequency of larger LD (**B**). Maximum diameters of lipid droplets (LD) were determined from 3 independent experiments. Results were obtained from 208 to 301 LDs per group. (****) p < 0.0001 by Student’s *t-*test. Scale Bar: 20 µm.

**Figure 6:**
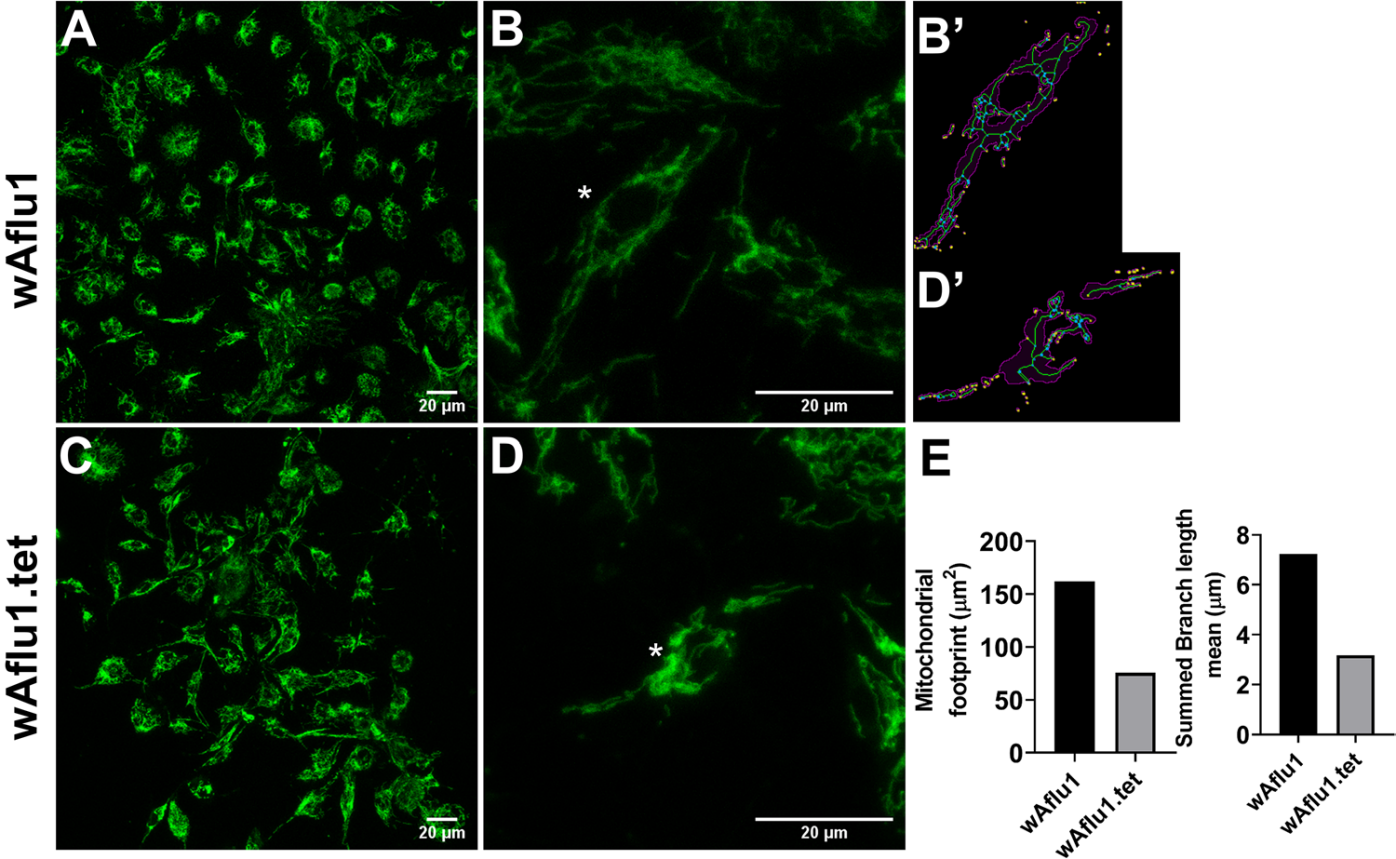
Mitochondrial staining on *Aedes fluviatilis* cell lines. Mitochondrial staining on *Aedes fluviatilis* cell lines wAflu1 (Wolb+) and wAflu1.tet (Wolb−) was detected using MitoTracker specific staining (**A**, **C**), respectively. Mitochondria change distribution in presence of Wolbachia showing more diffuse marking in wAflu1 (**B**) than wAflu1.tet (**D**) cell culture. The (**B′**, **D′**) represent the analysis of the cell indicated by an asterisk in (**B**, **D**) and magenta region is the binarized signal used for calculating the area (**E**). The green lines are the morphological skeleton and yellow dots represent the end points of the skeleton and the blue dots represent the junctions. Mitochondrial mass was higher in wAflu1 cells than in wAflu1.tet inferred by mitochondrial footprint and summed branch length mean (**E**). Scale Bar: 20 µm.

Proteins and glycogen are more abundant (by 15% and 70%, respectively) in *w*Aflu1 than in Aflu2 or wAflu1.tet cells (Fig. 4A,B and Supplementary Figure 3A and B, respectively), whereas Aflu2 cells present 20% more triglycerides than *w*Aflu1 (Fig. 4C). Accordingly, lipid droplets have higher diameters in wAflu1.tet than in *w*Aflu1 cells (Fig. 5A,B), with similar proportions of 3 and 4 µm lipid droplets, while the *w*Aflu1 cells showed a higher proportion of the 3 µm ones (Fig. 5B). When stained with Oil Red O, lipids seemed more abundant in Aflu2 cell line than in *w*Aflu1cells (Supplementary Figure 4). Additionally, *w*Aflu1 (Fig. 6A,B) cell line seems to have higher mitochondrial mass compared to wAflu1.tet (Fig. 6C,D) cell line, as detected by MitoTracker staining, and determined using mitochondrial footprint and mean branch length (Fig. 6B′,E, respectively). Aflu2 cells show the same pattern observed for wAflu1.tet (Supplementary Figure 5). Furthermore, the respiratory rate it seems to be more pronounced to wAflu1.tet cell culture in comparison to the cells with Wolbachia wAflu1 (Supplementary Figure 6).

### Aedes fluviatilis embryonic cells, with or without Wolbachia, respond to stimulation of the immune system

**Figure 7:**
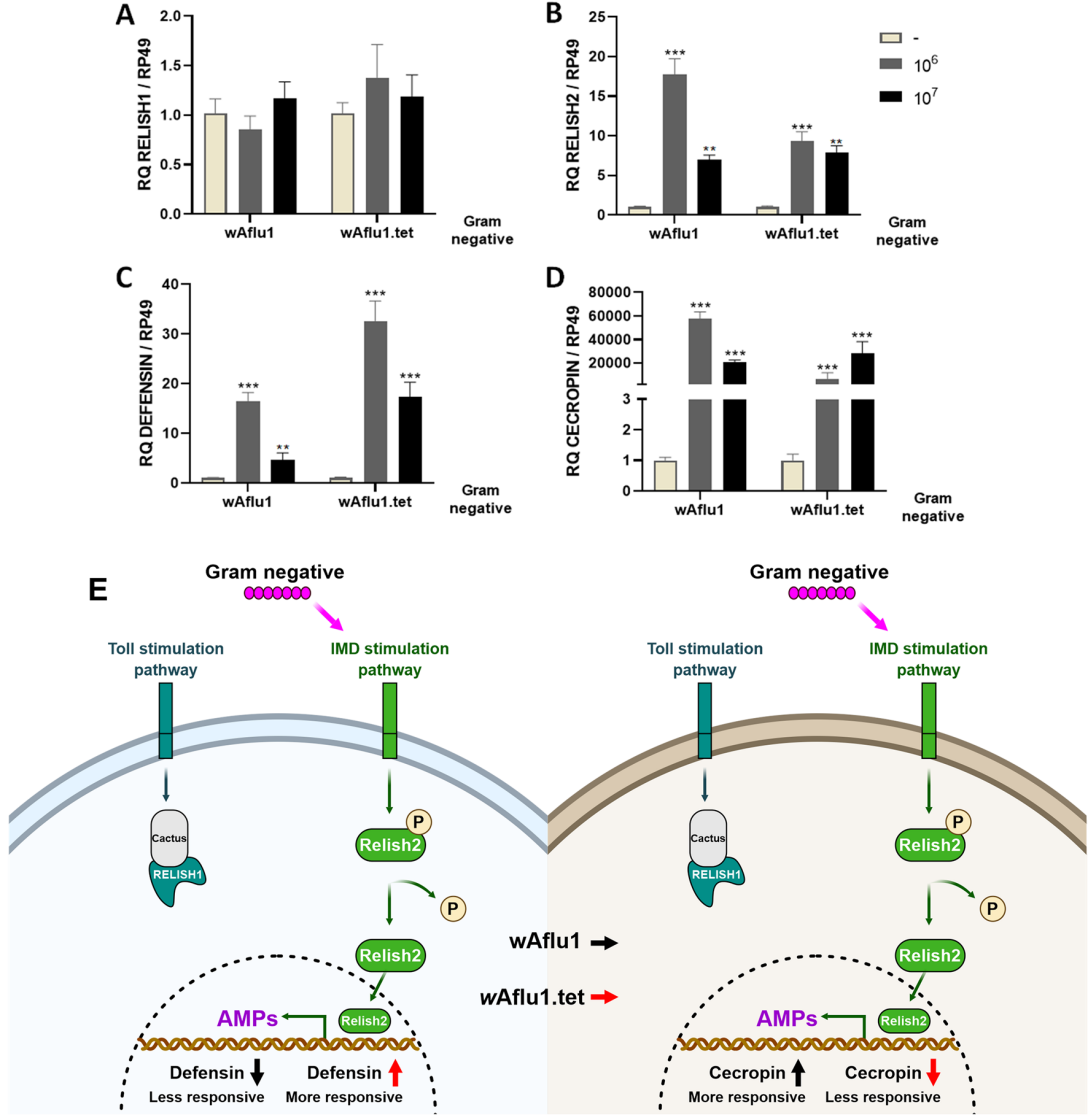
Innate immunity was activated by heat-killed Gram-negative (*Enterobacter cloacae*) bacteria.wAflu1 and wAflu1.tet cell cultures were challenged for 24 h with 10^6^ or 10^7^ heat-killed Enterobacter cloacae cells, then used for relative mRNA quantification of Relish 1 (**A**), Relish2 (**B**), defensin (**C**) and cecropin (**D**). Schematic representation (Created with BioRender.com) summarizing the different responses of *A. fluviatilis* cells to heat-killed Gram-negative *E. cloacae* (**E**). mRNA levels are expressed as mean ± SD (n = 3). Quantitative PCRs were statistically analyzed by ANOVA followed by Dunnett’s multiple comparison test. Asterisks indicate significant differences (**p < 0.01; ***p < 0.001).

Immune responses related to Toll and *Immune* Deficiency (IMD) pathways, as well as antimicrobial production, were evaluated in *w*Aflu1 and *w*Aflu1.tet cell cultures after exposure to heat-killed bacteria. *A. fluviatilis* cell cultures were incubated with 10^6^ or 10^7^ heat-killed Gram-negative (*Enterobacter cloacae*) bacterial cells. As expected, was not observed an increase in mRNA levels of Relish1 (Rel1) transcription factor responsive to Toll pathway in both cell lines (Fig. 7A). At the lowest bacterial concentration tested, *w*Aflu1 showed a significant increase in mRNA levels of Relish 2 (Rel2), transcription factor responsive to IMD pathway, with a 15- to 20-fold increase compared to control (Fig. 7B). While an 8- to tenfold increase Rel2 was observed in *w*Aflu1.tet cells in the stimulus with 10^6^ or 10^7^ heat-killed bacterial concentrations (Fig. 7B). The levels of mRNA for the antimicrobial peptide Defensin increased 20-fold in *w*Aflu1 line, while in *w*Aflu1.tet cells the Defensin increase was 30-fold (more responsive) (Fig. 7C). Interestingly, there was a 20.000- to 60.000-fold increase in mRNA levels for the antimicrobial peptide cecropin in *w*Aflu1 cells (more responsive) and a 20.000- to 40.000-fold cecropin increase in *w*Aflu1.tet cells (Fig. 7D). These data indicate that the two cell lines respond to immunological stimulus by increasing Relish2, cecropin and defensin expression. Figure 7E shows a schematic representation (Created with BioRender.com) summarizing the different responses of *A. fluviatilis* cells to heat-killed Gram-negative *E. cloacae.*

## Discussion

*Wolbachia pipientis* is a widespread endosymbiotic alphaproteobacterium that attracts research interest due to its effects on several signaling pathways, including metabolic and fitness improvement in different hosts^28,30^. The relationship between symbiosis and some biological processes, such as carbohydrate and lipid metabolic regulation and immune responses, are difficult to study in vivo. Thus, the establishment of new cell lines is an important and sought-after resource for cell and molecular biology studies, as well as the development of biotechnological products. Arthropod cell culture represents a fundamental tool in various areas of basic biological research, and have gained increasing interest in biotechnology, including cells lines from *Bombyx mori, Mamestra brassicae, Spodoptera frugiperda, Trichoplusia ni, Drosophila melanogaster* and others^31–36^. Cell biology and pathology studies which were highly dependent on in vitro studies with vertebrate species can now be partially conducted using invertebrate cell lines.

Here, we establish and characterize two novel cell lines (*w*Aflu1 and Aflu2) originated from *Aedes fluviatilis embryos*. Each cell line was isolated from embryonic tissue fragments of a single egg mass, and they were not cloned, therefore the number of different cell types that are present is unknown. The specific tissue of origin and level of differentiation of the cells comprising these lines are also unknown. The species identification of both lines was confirmed by partial sequencing of the 16S rRNA gene (Fig. 1A). *w*Aflu1 cells, derived from embryos naturally infected with Wolbachia, contained the expected bacterial strain as confirmed by WSP gene fragment sequencing (Fig. 1B). Aflu2 in turn, derived from embryos layed by female without Wolbachia, was confirmed as a Wolbachia-free cell line as well as wAflu1.tet originated from treatment of wAflu1 with tetracycline (Fig. 2A, lane c and Supplementary Figure 1). Nuclei staining with DAPI also offered additional evidence of Wolbachia presence in the cytosol compartment of *w*Aflu1 cells (Fig. 2B, enlarged left side panel). White^37^ previously used DAPI staining in host cell cytoplasm to demonstrate Wolbachia density. The presence of Wolbachia (wAflu1) affected cell morphology, with more peripheral and regular actin distribution (Fig. 3, red arrow head and zoomed image).

The bacteria also affected cell growth, *w*Aflu1 cells show a relatively fast proliferation rate (doubling time 3 days) compared with Aflu2 cells (doubling time 3.3 days) (data not shown). The same is observed for wAflu1.tet, presenting a relatively lower proliferation after 10 days compared with *w*Aflu1 cells (data not shown). In embryonic cell lines from other insects, the doubling time is approximately 2.5 days in *Bombyx mori*^38^ and approximately 1.75 days in *A. aegypti* Aag2^39^. The suggestion of wAflu1 cells grow faster than the Aflu2 indicate that the bacteria could act to improve the metabolic and nutritional status of the cell and future studies can clarify this phenomena. The identification of candidate metabolites or mechanisms to explain the endosymbiotic nature of this interaction is currently a great challenge for many research groups^40^. Newton and Rice^28^ bring a critical review suggesting that Wolbachia can improve host fitness with metabolic and nutritional support. These cell lines are intended to serve as models to understand primary and secondary metabolic pathways, including the nutritional host-bacteria interactions, as observed between *A. fluviatilis* and Wolbachia.

In this context, the major energy sources were measured in the new cell lines to assess different metabolic networks. The triglycerides content was 20% lower in *w*Aflu1 cells, showing a change in cell metabolism likely induced by Wolbachia, and suggesting that the host's metabolism is altered to use more lipids and the bacteria may benefit to use some metabolic intermediate (Fig. 4C). Wolbachia presents highly conserved pathways for fatty acid synthesis but depends highly on transport of carbon sources such as glycerate-3-phosphate^40^. Insects can synthesize fatty acids and other lipid classes, but are also sterol auxotrophs^41^, increasing the potential for competition for dietary cholesterol. Cholesterol modulation by Wolbachia is believed to have a major role during dengue virus infection in *Drosophila melanogaster*, where it was shown that increased dietary cholesterol reduced viral refractoriness in flies infected with wMel Wolbachia strain^42^, but the relevance of these observations in mosquitoes remains an open question. Similar effects were observed in *Aedes albopictus* Aa23 cell line, which showed a decreased lipid content in the presence of Wolbachia^43^. Our results show that Wolbachia-free cells (wAflu1.tet) have lipid droplets in the cytoplasm with higher diameter compared to mosquitoes with Wolbachia (wAflu1) (Fig. 5A,B). The higher relative frequency of lipid droplets of 4 µm diameter is observed in wFlu1.tet (Fig. 5B). On the other hand, in the presence of Wolbachia (wAflu1) the lipid droplet distribution changes, with a reduction in 4 µm lipid droplets abundance (Fig. 5B). In Aflu2 cell line, the lipids are more abundant compared to *w*Aflu1cells (Supplementary Figure 4). These results suggested that the presence of Wolbachia makes the mosquito cells mobilize more lipids compared to Wolbachia-free cells, as maintenance of Wolbachia cellular metabolism possibly creates more demand for this energy source, although it cannot be ruled out the possibility that the lower lipid levels are due to decreased lipid synthesis, also as a consequence of Wolbachia infection. Other studies on these metabolic pathways are required to understand how Wolbachia affects the availability of substrates in these cell conditions.

Protein content, in turn, is 15% lower in Aflu2 and wAflu1.tet compared with *w*Aflu1 cells (Fig. 4A and Supplementary Figure 3A). Notably, in *A. aegypti,* Wolbachia has been observed to act as a parasite, competing with the host for amino acids derived from protein degradation^44^. Our results indicate that the opposite takes place in the symbiotic interaction between Wolbachia and *A. fluviatilis* mosquito, with increased protein content when Wolbachia is present (*w*Aflu1) (Fig. 4A and Supplementary Figure 3A). In this case, the bacteria might improve host energy performance by an unknown mechanism. Caragata^44^ also observed an increase in fecundity and egg viability in *A. aegypti* infected with wMelPop strain of Wolbachia after a diet composed of amino acids and sucrose, highlighting that not only amino acids but also carbohydrates are involved in this parasite-host interaction.

Glycogen is the major carbohydrate reserve in animals^45^. Previous work by our group has illustrated how insect energy metabolism can be modulated by Wolbachia to obtain energy. da Rocha Fernandes^46^ showed that during *A. fluviatilis* embryogenesis, glycogen level increased gradually up to 24 h, followed by a decline at 48 h. Interestingly, the peak of glycogen concentration at 24 h was about 3 times higher in Wolbachia-infected embryos than in Wolbachia-free embryos, showing that the bacteria could be stimulating glycogen synthesis by the host cells^46^. In the present work, the results support this conclusion, showing that glycogen content is higher in the presence of Wolbachia (*w*Aflu1 cells) compared with Aflu2 and wAflu1.tet cells (Fig. 4B and Supplementary Figure 3B). Interestingly, GSK-3 gene silencing increased glycogen and the number of bacteria in *A. fluviatilis* embryos, compared to control^46^, further evidencing the complex nature of this host-symbiont interaction in the context of carbohydrate metabolism and energy regulation. Genes encoding enzymes involved in the synthesis and degradation of glycogen are absent in the Wolbachia genome, as are the genes for enzymes of the glycolytic preparatory phase^46^ (genome data available at http://tools.neb.com/wolbachia/. Accordingly, it was suggested that Wolbachia may be able to internalize host pyruvate, which would be further processed by the bacterial pyruvate phosphate dikinase^46^. Pyruvate phosphate dikinase uses pyruvate to produce fructose 6-phosphate and acetyl-CoA, as described by^47^. Recently, Cariou^48^ showed that Wolbachia can induce indirect selective sweeps on host mitochondria, to which they are connected within the cytoplasm. The resulting reduction in effective population size might lead to smaller mitochondrial diversity and reduced efficiency of natural selection. In the context of our newly developed cell lines, mitochondrial staining (Fig. 6A–D and Supplementary Figure 5) suggests that cells with Wolbachia have distinct profile in the distribution of mitochondria and highly footprint and longer mean branch lengths compared to Wolbachia-free wAflu1.tet cells (Fig. 6E), suggesting an increase in mitochondrial mass by the host when Wolbachia is present. Surprisingly, in respirometry (oxygen consumed for ATP synthesis) it was observed that wAlfu1.tet cells have a higher respiratory rate compared to wAflu1 cells (Supplementary Figure 6). Therefore, the greater mitochondrial mass observed in the presence of Wolbachia suggests other metabolic needs of these cells in addition to ATP synthesis, such as the synthesis of metabolic intermediates that transfer carbon to gluconeogenesis or to the metabolism of amino acids and fatty acids. Also, it has not been shown that Wolbachia uses oxygen as the final acceptor in the electron transport chain. Thus, our model can be a powerful tool to study this context. Greater cell proliferation, greater amounts of glycogen and protein, and lower oxygen consumption in wAflu1 cells indicate a key role for Wolbachia in this relationship.

When both cell lines were challenged by Gram-negative (*E. cloacae*) heat-killed bacteria, the expression of Relish1 not change and Relish2 increased, suggesting IMD pathways activation (Fig. 7A,B). As well as Toll, Janus kinase signal transducer and activator of transcription (JAK-STAT) pathways, the IMD pathway players an important role on transcription of antimicrobial peptides (AMPs)^49^. Antimicrobial peptides such as cecropins are related to depletion of both Gram negative and Gram positive bacteria^50,51^. Aag2 cells (*Aedes aegypti* Mosquito Embryonic Cells) infected with DENV virus become less able to transcribe cecropin and defensin and more susceptible to infection by Gram-positive and Gram-negative bacteria^49^. On the other hand, in *Aedes aegypti* Aag2 cell line, naturally free of Wolbachia, defensin production is highly activated by challenge with heat-killed *E. cloacae*^52^. In our results, cecropin was strongly up-regulated in both cell lines, with or without Wolbachia, indicating that both cell lines were able to respond to the immune stimulus. Interestingly, cecropins was 20,000-fold more expressed in *w*Aflu1 cells compared to *w*Aflu1.tet cells. Furthermore, defensin was 10-folds more expressed in *w*Aflu1.tet cells if compared to *w*Aflu1 cells (Fig. 7C). This dataset suggests that Wolbachia may change the profile of the transcriptional of the antimicrobial peptides described here.

The capacity of *Aedes fluviatilis* to transmit yellow fever and dengue virus has been demonstrated experimentally, moreover, it is a vector of *Plasmodium gallinaceum*. Recently, was reported *A. fluviatilis* infected by chikungunya and this demonstrate a potential specie that can be responsible by future epidemies, such as *A. aegypti* and *A. albopictus* species^53,54^. The novel cell lines described here can contribute to understanding host-symbiont interaction and the mutual metabolic regulation between *A. fluviatilis* and Wolbachia, in addition to being useful in research strategies to control dengue and other arboviruses. The results presented here also indicate that the natural symbiotic relationship between Wolbachia and *A. fluviatilis* involves modulation of the host metabolism and immunity. In summary, we provide cellular and molecular characterization of two novel cell lines (*w*Aflu1 and Aflu2), with potential to be used in investigations of pathogen/vector immunological interactions and symbiotic relationships, as well as in applied research to prevent insect-transmitted pathogens.

## Materials and methods

### Establishment and maintenance of wAflu1 and Aflu2 cells

*w*Aflu1 cells were derived from *A. fluviatilis* embryos naturally infected with the *w*Flu Wolbachia strain, whereas Aflu2 cells were derived from embryos of *A. fluviatilis* previously treated with tetracycline for three generations to remove the Wolbachia infection (wFluTet)^23,55^ and subsequently kept free of Wolbachia for at least 20 generations without tetracycline treatment. The primary culture was made in September 2019 using eggs pooled 6–24 h after the onset of oviposition. The eggs laid by females of naturally infected *A. fluviatilis* with the *w*Flu Wolbachia strain or Wolbachia-free wFluTet *A. fluviatilis* were kept overnight at 4 °C on sterile water, followed by incubation for 2 h at 37 °C to stimulate cell division^56^. Approximately 20–40 mg eggs were mechanically separated and surface-sterilized by immersing three times for 3 min in 95% ethanol and once for 5 min in biocide hypochlorite detergent [10,000 p.p.m. available chloride (Cl^−^)] containing 1% tween 20. Eggs were then washed three times in sterile phosphate buffered saline PBS, pH 7.4 (137 mM NaCl; 10 mM Na_2_HPO_4_; 2.7 mM KCl; 1.8 mM KH_2_PO_4_) and once in L-15 complete medium consisting of Leibovitz's L-15 medium (Sigma, #L4386) supplemented with amino acids, glucose, mineral salts, vitamins (concentrated medium) and then, immediately before use, diluted 3:1 in sterile water and supplemented with 10% tryptose phosphate broth (Sigma, #T8782), 10% heat-inactivated fetal calf serum (Gibco), and commercial antibiotic penicillin–streptomycin (Sigma P4333, 1:500), according to Munderloh and Kurtti^57^. Using sterile Glass/Teflon Potter Elvehjem homogenizers, eggs were crushed in 500 μL of L-15 complete medium. Homogenized embryos were transferred to a 25 cm^2^ culture bottle with 5 mL fresh L-15 complete medium at pH 6.8–7.2, and incubated at 28 °C for cell culture establishment. To reduce pH fluctuations, half of the culture medium volume was exchanged for fresh medium every 2–4 days. After cell colonies were first observed (about 3–4 weeks), the culture medium was completely replaced weekly. This process removed most remaining embryo fragments, whereas cells attached to the surface grew to form a monolayer (3–4 months). Both cell lines, *w*Aflu1 and Aflu2, were subcultured for the first time in the fifth month by scraping part of the cells with a serological pipette and transferring them to a new flask with 5 ml of fresh medium and the other subcultures were made using 5 mL syringe with needle to detach the cells. These two established lineages have been maintained in L-15 complete medium^57^.

### Microscopy

*w*Aflu1 or Aflu2 cells from the 4th–5th passage was seeded at an initial concentration of 5 × 10^6^ cells per bottle (25 cm^2^) in 5 mL medium to be used seven days later at an expected concentration of 10^7^ cells per bottle (2 × 10^6^ cells/mL), approximately. Intact live cells were observed by microscopy using nuclear staining (Hoechst), mitochondria (Mitotraker Green) and Lipid droplets (Nile red). A sample with 1 × 10^5^ cells was seeded in 2 mL of medium in cell culture dish and incubated in complete medium at 28 °C for 3 days. Hoechst 33,342 [1/1000 (10 mg/mL)] was added for 10 min after which cells were observed under phase contrast (ph) and fluorescence imaging using a Leica DMI4000 B microscope. For mitochondrial staining, Mitotracker Green (Thermofisher) solution (200 nM) was added for 30 min at 28 °C, followed by washing in PBS at least twice and imaged in a Leica TCS-SPE laser scanning confocal microscope (Leica Microsystems). For analysis of lipid droplets, cells were stained with Nile Red and DAPI (Sigma-Aldrich, Saint Louis), as previously described^58^. The medium was removed and cells incubated for 5 min in 1 mg/ml Nile Red and 2 mg/ml DAPI made up in 75% glycerol and immediately imaged in a Leica TCS-SPE laser scanning confocal microscope (Leica Microsystems). The excitation wavelengths used were 543 nm for Nile Red and 280 nm for DAPI. The average diameters of the lipid droplets were obtained from three images from each group, in three independent experiments, using the DAIME image analysis software, after edge detection automatic segmentation^59^. The lipid droplets diameters were plotted in a frequency histogram (bin width = 1).

Alternatively, 5 × 10^5^ cells were seeded on round coverslips in a 24-well plate in 500 µL of L-15 complete medium per well and fixed for subsequent staining of actin and nuclei. After 3 days of growth, cells were washed twice with preheated PBS pH 7.4 and fixed in 4% paraformaldehyde solution in PBS for 10 min at room temperature, followed by two PBS washes. Then, cells were permeabilized with 0.1% Triton X-100 in PBS for 3 to 5 min and washed two times with PBS. To reduce nonspecific background staining, 1% bovine serum albumin (BSA) was added for 30 min and washed with PBS before staining. Staining was carried out with 165 nM actin staining N354 NBD phallacidin (Invitrogen) for 20 min at room temperature. Finally, cells were air dried, mounted in a permanent mount (Vectashield), and stored in the dark at 2–6 °C. For nuclear staining, after fixed in 4% paraformaldehyde solution in PBS for 10 min at room temperature and washed with PBS, a volume of 300 nM DAPI stain solution sufficient to cover the cells was added and incubated for 5 min, protected from light. Staining solution was removed and cells were washed 2–3 times in PBS. Fixed stained cells were observed in a Laser Scanning Confocal Microscope, model Zeiss LSM710.

### Cryopreservation and defrost

To the cryopreservation protocol, 1-mL cryotubes were maintained in a Mr. Frosty container (Nalgene Thermo Scientific) pre-cooled at—80 °C. About 5 × 10^6^ cells were centrifuged at 200 × g for 5 min and the cell pellet was gently resuspended in 1 mL freezing solution containing fetal calf serum and 10% DMSO, keeping the tubes on ice. The 1-mL cell suspension was transferred to ice-cold cryotubes, placed in a Mr. Frosty container at − 80 °C freezer for 24 h, and then in liquid nitrogen. For defrost, the cryotubes were thawed rapidly by immersion in a 37 °C water bath. The cell suspension was transferred to 9 mL of culture medium and centrifuged at 200 × g for 5 min. The pellet was resuspended in fresh medium, transferred to a 25 cm^2^ bottle, and maintained at 28 °C for recovery. In addition, to confirm the viability of intracellular Wolbachia after cryopreservation and thawing protocols, the recovered cells were used for RNA extraction and RT-PCR analysis of WSP transcription using the specific primers described below.

### RNA extraction, cDNA synthesis, and sequencing

Total RNA was extracted from 5 × 10^5^ cells, using Trizol Reagent (Invitrogen) according to the manufacturer's instructions. RNA concentration and purity of all samples were determined on a NanoDrop spectrophotometer (Thermo Scientific). cDNA was synthesized from 2 µg of total RNA using the High-Capacity cDNA Reverse Transcription kit (Applied Biosciences).

cDNA obtained from *w*Aflu1 or Aflu2 cell lines was used for PCR amplification of 16S gene using primers designed based on the ribosomal 16S sequence found in the transcriptome of the mosquito *A.fluviatilis* and deposited in GenBank under the accession number MW574133 (forward 5′-CCCACTGAAATTTTAAAGGGCCGC-3′ and reverse 5′-CGCCGGTTTGAACTCAGATCATGTA-3′). WSP gene (GQ917108) was amplified using the following primers: forward 5′-TGGTCCAATAAGTGATGAAGAAAC-3’ and reverse 5′-AAAAATTAAACGCTACTCCA-3′^60^. PCR products were analyzed by 1% agarose gel electrophoresis with ethidium bromide staining, and the amplified fragments were purified using the Wizard SV Gel kit (Promega). The samples were sequenced by Standard sequencing based on the Capillary Electrophoresis Sequencing (CES) automation system (Macrogen Service) and analyzed using Bioedit software version^61^ 7.2.5.

### Glycogen, triglycerides, and protein quantification

For quantification of different energy sources, 5 × 10^6^ cells were lysed in 200 µL Tris/HCl buffer pH 7.4 containing 0.1% triton × 100. For glycogen content determination, 30 µL lysed cells were incubated with 200 mM sodium acetate, pH 4.8, containing 1 unit of alpha-amyloglucosidase (Sigma Chemicals) for 4 h at 40 °C, after which glucose concentration was measured by the glucose oxidase method using a commercial kit (Labtest Ref.133). Free glucose measured in samples without alpha-amyloglucosidase was subtracted from the results, and a standard curve was generated to calculate glycogen levels in the samples.

Triglycerides were quantified using an adapted colorimetric enzyme method kit (Ebram). Lysed cells (80 µL) were added to 1 mL of color reagent (triglyceride kit buffer), and the reaction mixture was incubated for 15 min at 37 °C. Absorbance was measured at 500 nm using a spectrophotometer (Multiskan Go Thermo Scientific), and triglyceride concentration (normalized by 10^5^ cells) was calculated according to the kit’s recommendation.

Protein content was determined using the BCA method with bovine serum albumin (BSA) as a standard, according to manufacturer’s recommendations (Sigma Chemicals). All results are presented as mean and standard deviation from three independent experiments^62^.

### Analysis of the mitochondrial footprint and mean branch lengths

Image was opened on Fiji^63^ and a single cell was selected using the rectangular ROI tool, preprocessed^64^ and converted in 8-bit single channel. The script by navigating to Plugins > StuartLab > MiNA Analyze Morphology was performed^63,64^. An overlay was generated to visually inspect the faithfulness of the analysis. Then a copy of the image with overlays was save by flatten the image and save it in tiff format.

### Immune challenge with heat-killed bacteria and qPCR analysis

To analyze the effect of immune stimulation on *w*Aflu1 and wAflu1.tet cultures, 5 × 10^5^ cells were seeded in 24-well plates with 500 µL L-15 complete medium. After three days, the cells were exposed to 10^6^ or 10^7^ Gram-negative (*Enterobacter cloacae)* heat-inactivated bacteria during 24 h. Total RNA extraction and cDNA synthesis were carried out as described in “RNA extraction, cDNA synthesis, and sequencing” section. Specific primer pairs were designed to perform quantitative PCR to assess Toll pathway response using Relish1 (Rel1) gene (MW574128; forward 5′-CAGCCAATCAGCAACAGAAA-3′ and reverse 5′-ATTCGTTTGATGGGCGATAG-3′), or IMD pathway response using Relish2 (Rel2) gene (MW574129; forward 5′- ATTGTTTCCGTCTGGATTCG-3′ and reverse 5′- TCACGCAGAACGTATGAAGC-3′). Also, the relative expression of antimicrobial peptides was investigated using specific primers for Defensin gene (MW574130; forward 5′-AACTCTCCTCTCACGCCGTA-3′ and reverse 5′-TACGAGCGAACATCATCAGC-3′) and Cecropin gene (MW574131; forward 5’- CGATTTGACGTTCGAGATGA-3′ and reverse 5′-CGCCGGTTTGAACTCAGATCATGTA-3′).

qPCRs were carried out on Applied Biosystems StepOne platform, in 20 μL, with 20 ng of cDNA, 500 nM of each primer (final concentration) and qPCRBIO SyGreen Mix HI-ROX (PCR BIOSYSTEMS), following the manufacturer’s recommendations. Relative transcription was determined using the Ct values from each run on Relative transcription Software Tool—REST^52^. The constitutively expressed gene of the ribosomal protein RP49 (MW574127) was used as a reference gene with set primers forward 5′- GATGCAGAACCGTGTCTACT-3′ and reverse 5′- AGCTTACTCGTTTTCTTGCG-3′).

### Respirometry analysis in both wAflu1 and wAflu1.tet cell cultures

Respiratory activity was performed using a two-channel titration injection respirometer (Oxygraph-2 k, Oroboros Instruments, Innsbruck, Austria), calibrated with Schneider′s medium at 28 °C. Cells were harvested, counted and 2 × 10^6^ cells were suspended in 300 μL of Schneider′s medium. Both wAflu1(Wolb+) and wAflu1.tet (Wolb−) cell lines were placed into the O2K chamber in a concentration of about 1 × 10^6^/mL and were incubated with continuous stirring at 750 rpm for about 10 min. The analysis was performed at 28 °C and 750 rpm. The protocol was started by adding 250 ng/mL oligomycin, and the oxygen consumption coupled to ATP synthesis (ATP-linked respiration) was calculated by subtracting the oxygen consumption after the addition of the inhibitor of ATP synthase from basal respiration rates. The maximum uncoupled respiration was induced by stepwise titration of carbonyl cyanide p-(trifluoromethoxy) phenylhydrazone (FCCP) to reach final concentration of 300 nM. Next, respiratory rates were inhibited by the injection of 0,5 μg/mL antimycin A. The residual oxygen consumption (ROX) represents the oxygen consumed by the cells, not due to respiration. The maximum respiratory rates (ETS) were calculated by subtracting the antimycin resistant oxygen consumption from FCCP-stimulated oxygen consumption rates. The spare capacity is the amount of extra ATP that could be produced by oxidative phosphorylation in case of increase in energy demand that was calculated by subtracting FCCP-stimulated oxygen consumption rates from basal respiration rates. The leak respiratory states, that represents the oxygen consumption in presence of substrates but in the absence of ATP synthesis, was calculated by subtracting the oligomycin from the antimycin resistant oxygen consumption. All oxygen consumption rates were normalized per 1 × 10^6^ cells/mL.

## Supplementary Information

Supplementary Information.

## Data Availability

*Aedes fluviatilis* sequences were deposited in the NCBI with GenBank accession numbers for rRNA 16S (MW574133), gene encoding ribosomal protein RP49 (MW574127), Relish1 (MW574128), Relish2 (MW574129), Defensin (MW574130) and Cecropin (MW574131).
