# Supplementary material for: Aedes fluviatilis cell lines as new tools to study metabolic and immune interactions in mosquito-Wolbachia symbiosis
Source: Sci Rep. 2021 Sep 28;11:19202. doi: 10.1038/s41598-021-98738-7 (PMC8478883; doi:10.1038/s41598-021-98738-7)
Supplement: Supplementary file 1 — Supplementary Information. [file 41598_2021_98738_MOESM1_ESM.pdf]

Aedes fluviatilis cell lines as new tools to study metabolic and immune interactions in mosquito-Wolbachia symbiosis

Christiano Calixto Conceição[a,c], Jhenifer Nascimento da Silva[a,c], Angélica Arcanjo[a,c], Cíntia Lopes Nogueira[a,c], Leonardo Araujo de Abreu[a,c], Pedro Lagerblad de Oliveira[a,c], Katia C. Gondim[a,c], Bruno Moraes[a,c], Stephanie Serafim de Carvalho[a,c], Renato Martins da Silva[a,c], Itabajara da Silva Vaz Jr.[b,c], Luciano Andrade Moreira[d,c] and Carlos Logullo[#a,c]

[a] Laboratório de Bioquímica de Artrópodes Hematófagos, Laboratório de Bioquímica e Fisiologia de Insetos – IBqM and Laboratório Integrado de Bioquímica Hatisaburo Masuda – NUPEM, Universidade Federal do Rio de Janeiro - UFRJ, RJ, Brazil;

[b] Centro de Biotecnologia and Faculdade de Veterinária, Universidade Federal do Rio Grande do Sul - UFRGS, Porto Alegre, RS, Brazil;

[c] Instituto Nacional de Ciência e Tecnologia em Entomologia Molecular, Rio de Janeiro, RJ, Brazil.

[d] Grupo Mosquitos Vetores: Endossimbiontes e Interação Patógeno Vetor, Instituto René Rachou - Fiocruz, Belo Horizonte, Minas Gerais, Brazil.

[#]Corresponding author: Carlos Logullo

Universidade Federal do Rio de Janeiro, Av. Carlos Chagas Filho, 373, bloco D, subsolo, sala 05. Prédio do CCS. Ilha do Fundão, Rio de Janeiro - RJ - 21941-902 – Brazil. Tel. +55 21 -39386751, carlos.logullo@bioqmed.ufrj.br

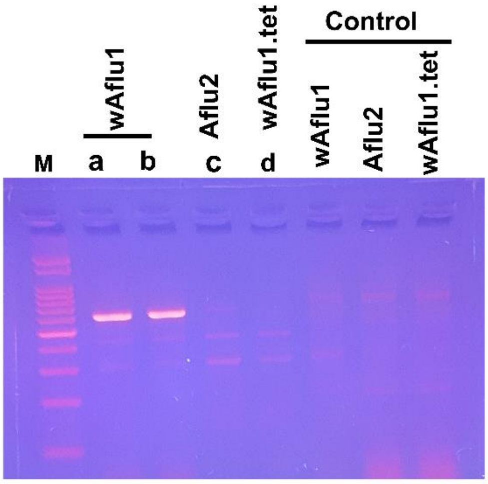

Supplementary Figure 1: Wolbachia confirmation in embryonic cell lines. *w*Aflu1 was positive for the Wolbachia-specific gene, WSP, which was absent in Aflu2 cells (lane a and c, respectively). Amplification of WSP gene transcripts was still detected in *w*Aflu1 cells after recovery of cryopreservation (lane b). Cell culture wAflu1.tet has Wolbachia removed by tetracycline treatment (lane d) All controls without reverse transcriptase has not WSP amplification. The WSP fragment amplified by RT – PCR has about 650 pb, as indicated by 100 pb marker (lane M).

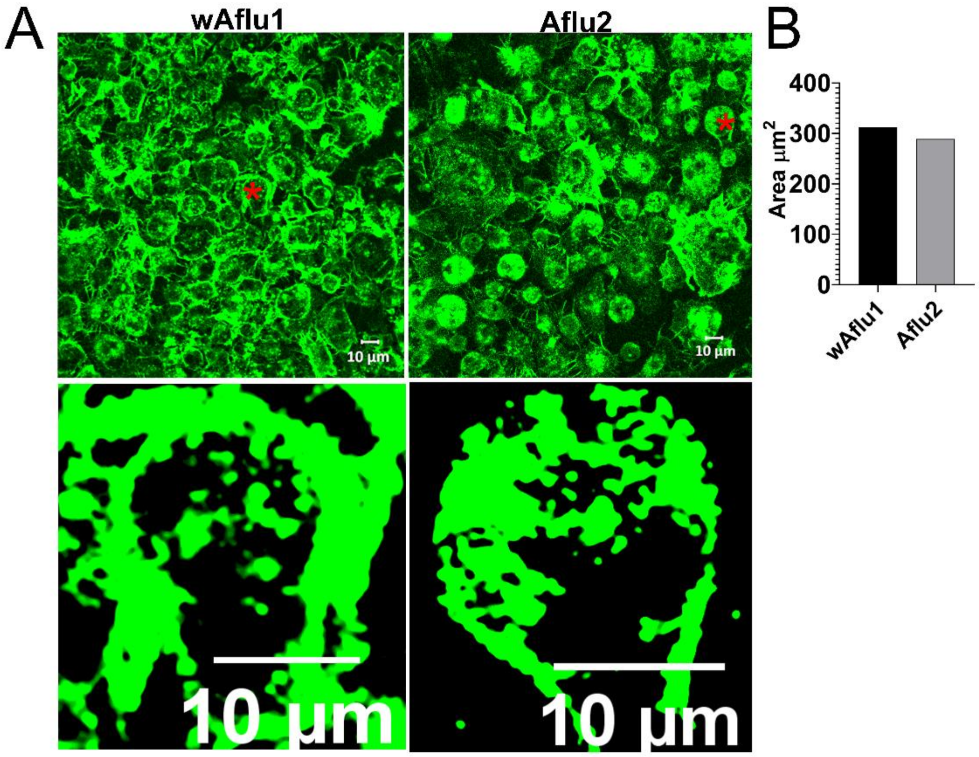

Supplementary Figure 2: *w*Aflu1 has a more peripheral actin distribution than Aflu2. (A) Actin cytoskeletal morphology (green) was observed three days after subculture using Phallacidin. wAflu1 cell line has more peripheral actin distribution than Aflu2 top panel and red asterisk indicating the enlarged cell (bottom panel). Scale Bar: 10 µm. (B) Enlarged cells represented in bottom panel had the area quantified demonstrating similar areas.

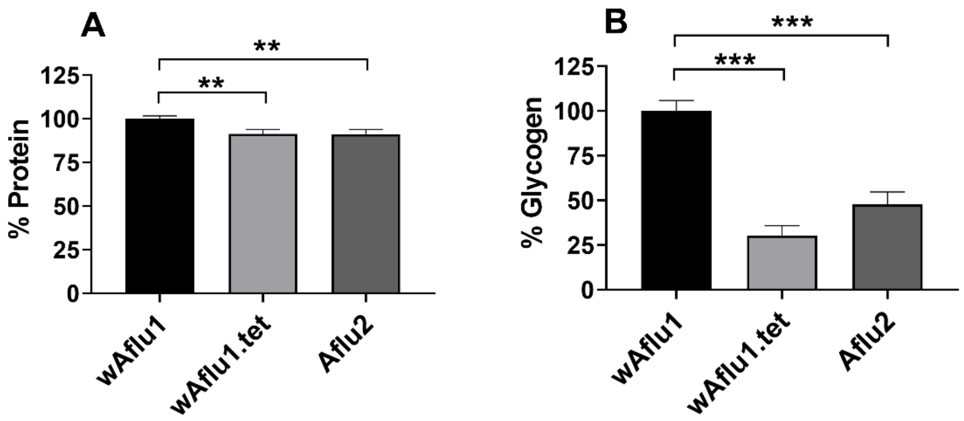

Supplementary Figure 3: Wolbachia change protein and glycogen on *Aedes fluviatilis* cells. wAflu1 cell line (Wolb+) has higher protein and glycogen contents in comparison to both Aflu2 and wAflu1.tet cell line without Wolbachia. The percentual was calculated from glycogen amount and normalized by 10^5 cells. The experiments were performed with three independent biological samples in three experimental replicates each, $^{**}p < 0.01$, $^{***}p < 0.001$, using student's t-test.

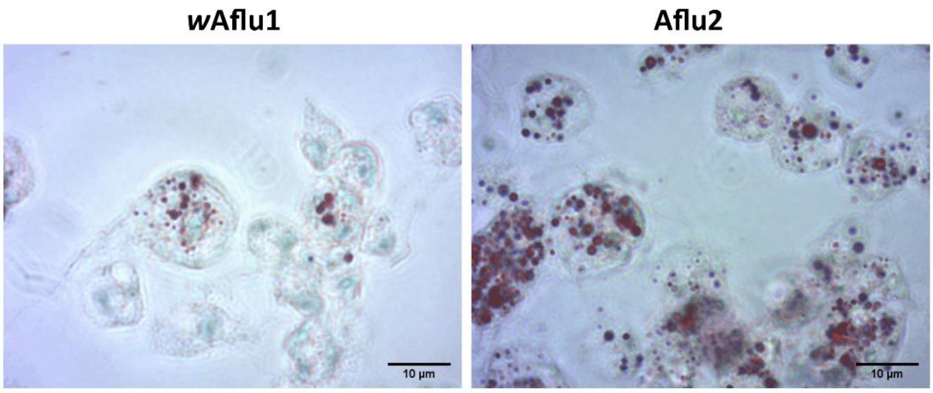

Supplementary Figure 4: Aflu2 cells have more lipid droplets than *w*Aflu1. Cell staining by Oil red showed more numerous lipid vesicles in Aflu2 cells line than in *w*Aflu1.Scale Bar: 10 µm.

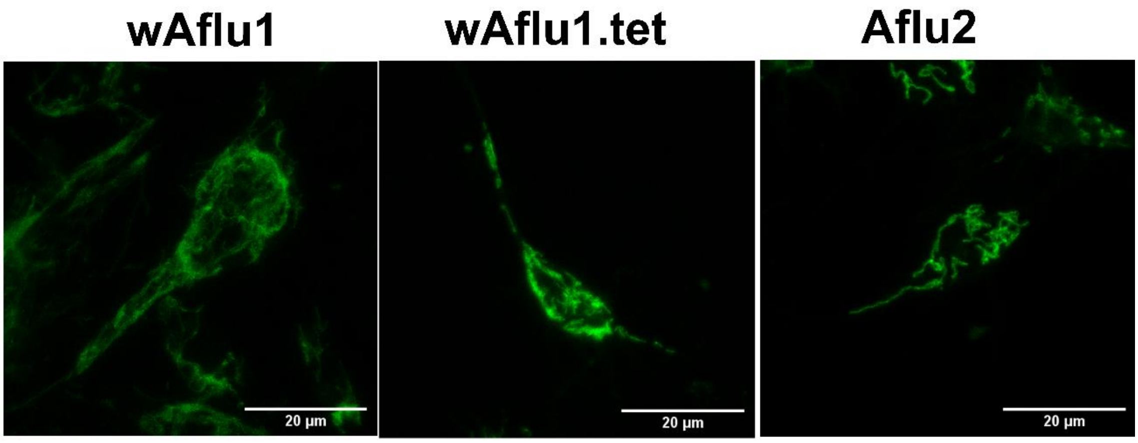

Supplementary Figure 5: Mitochondrial staining show different distribution patterns on *Aedes fluviatilis* cells. Mitochondrial staining on wAflu1 (Wolb+) and both wAflu1.tet and Aflu2 without Wolbachia was detected using MitoTracker specific staining. The mitochondria distribution in presence of Wolbachia exhibit a more diffuse marking in wAflu1 than wAflu1.tet and Aflu2. Scale Bar: 20 µm.

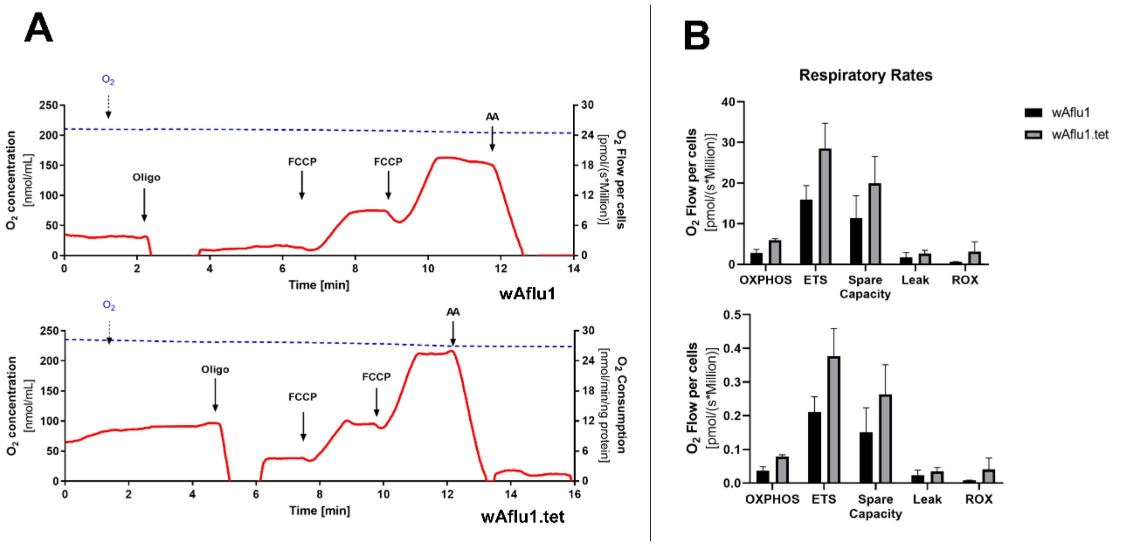

Supplementary Figure 6: Mitochondrial respiratory rates on wAflu1 and wAflu1.tet cells. A) Typical traces of oxygen consumption rates (OCR) of wAflu1 and wAflu1.tet cells. The blue line shows the oxygen concentration in the sealed chamber and the red line shows the OCR of the cells. Additions of OXPHOS modulators, oligomycin (oligo), FCCP, and Antimycin A (AA) are represented with arrows and their concentrations were reported in the methods section. B) Quantitative comparison of oxygen consumption rates (top panel) and normalized oxygen consumption rates by mitochondrial footprint (bottom panel). Data are expressed as pmoles/min/mL/10^6cells.
